# Linear-Nonlinear Switching Active Disturbance Rejection Speed Controller for Permanent Magnet Synchronous Motors

**DOI:** 10.3390/s22249611

**Published:** 2022-12-08

**Authors:** Ying Qu, Bin Zhang, Hairong Chu, Xiaoxia Yang, Honghai Shen, Jingzhong Zhang

**Affiliations:** 1 Changchun Institute of Optics, Fine Mechanics, and Physics, Chinese Academy of Sciences, Changchun 130033, China; 2 The University of Chinese Academy of Sciences, Beijing 100049, China; 3 Forest Protection Research Institute of Heilongjiang, Harbin 150040, China

**Keywords:** active disturbance rejection control (ADRC), linear-nonlinear switching active disturbance rejection control (SADRC), permanent magnet synchronous motor (PMSM), speed controller

## Abstract

To combine the advantages of linear active disturbance rejection control (LADRC) and nonlinear active disturbance rejection control (NLADRC) and improve the contradiction between the response speed and control precision caused by the limitation of parameter α in NLADRC, a linear-nonlinear switching active disturbance rejection control (SADRC) strategy based on linear-nonlinear switching extended state observer (SESO) and linear-nonlinear switching state error feedback control law (SSEF) is proposed in this paper. First, the reasons for the performance differences between LADRC and NLADRC are analysed from a theoretical point of view, then a linear-nonlinear switching function (SF) that can change the switching point by adjusting its parameters is constructed and then propose SESO and SSEF based on this function. Subsequently, the convergence range of the observation error of the SESO is derived, and the stability of the closed-loop system with the application of SSEF is also demonstrated. Finally, the proposed SADRC control strategy is applied to a 707 W permanent magnet synchronous motor (PMSM) experimental platform, and both the dynamic and static characteristics of SADRC are verified. The experimental results show that the proposed SADRC control strategy can well combine the performance advantages of LADRC and NLADRC and can better balance the response speed and control precision and has a better capacity for disturbance rejection, which has potential application in engineering practise.

## 1. Introduction

PMSMs are increasingly used in modern alternating current servo systems because of their high performance, light weight, and high power density [1]. The classical control of a PMSM is a cascade control structure using a proportional-integral (PI) controller, where PI controllers are used for the outer loop speed controller and inner loop current controller. PI controllers have the advantages of simple structure, high steady-state accuracy, and good stability and are widely used in linear time-invariant systems [2]. However, a PMSM is a typical nonlinear multivariable coupled system, accompanied by various uncertainty perturbations, such as external unknown load, internal friction, and nonlinear magnetic field, which makes it difficult for PI controllers to meet the requirements of higher control performance [3].

In recent years, research on high performance and high precision control has continuously expanded. Sliding mode control [4,5], model predictive control [6,7], iterative learning control [8], neural network control [9,10], fuzzy control [11,12], ADRC [13,14] and many other control algorithms have been proposed and improved and applied to PMSM control. These control algorithms have improved the control performance of PMSMs in various aspects. ADRC is widely used in various industrial applications because of its robustness and independence from the controlled object model [15].

ADRC was first proposed by Han [16] and later by Han’s collaborator Gao, who proposed a method for ADRC parameter tuning [17,18]. In recent decades, an increasing number of scholars have devoted themselves to the study of ADRC. Yang et al. applied the hyperbolic tangent function to the tracking differentiator (TD) of ADRC to simplify its structure, improve the tracking accuracy, and reduce the effect of load perturbations on the system [19]. Qu et al. proposed an improved LADRC through a correction of perturbation compensation and an improved expansion state observer (ESO). The tracking performance and dynamic stiffness of the LADRC were significantly improved [20].Qu et al. proposed an enhanced linear active disturbance rejection controller (ELADRC) consisting of two linear expansion state observers (LESOs) and a proportional current controller, and experimentally verified the effectiveness of the proposed ELADRC [21]. Shi et al. integrated extended state filters into the ADRC system for signal filtering, which solved the problems of time delay and noise [22]. Li et al. proposed a new control method based on NLADRC and proportional-integral control (PI). In this control framework, a feedforward control based on a nonlinear tracking differentiator (NTD) is designed to improve the tracking performance of the system. Experiments show that the method can better suppress the low-frequency mechanical resonance when applied to a large telescope [23]. In addition, some scholars have obtained ADRC with higher control performance by combining it with other advanced control algorithms. Qu et al. proposed a new sliding mode current controller based on active disturbance rejection. First, a fast response sliding mode controller was designed based on the upper bound of the internal disturbance. Then, an ESO was designed to estimate the internal disturbance of a PMSM in real time, and the estimated internal disturbance was used to update the control law of the sliding mode control in real time. The improved active disturbance rejection sliding mode current controller improves the steady-state and transient current tracking performance and enhances the robustness to internal disturbances [24]. Gao et al. proposed a compound control scheme that combines the advantages of a fractional-order proportional-integral-differential controller and LADRC. The compound control method was experimentally verified to have satisfactory performance in terms of rapidity and robustness [25]. Overall, ADRC has good control performance, but the tuning of its parameters is relatively complicated and lacks systematic theoretical support. In this case, research on ADRC parameter tuning is also necessary. In this regard, Lu et al. proposed a new dual-loop drive system based on position-speed integrated ADRC. A fuzzy parameter self-tuning method was proposed to solve the problem of poor load adaptation due to difficult ADRC parameter tuning [26].

Existing studies on ADRC reveal that LADRC has the advantages of easy parameter tuning and that the capacity of disturbance rejection does not degrade with the increase in the disturbance amplitude, while NLADRC has higher control precision [27]. Currently, studies have started to combine LADRC and NLADRC to exploit their respective performance advantages. Hao et al. proposed a hysteretic switching strategy to estimate and compensate for the total disturbance. In addition, a parameter tuning strategy for SADRC was given due to the limitation of switching conditions [28]. Lin et al. proposed a new SADRC class, which combines LESO and nonlinear extended state observer (NLESO) through the ESO observation error to enhance the robustness of a PMSM control system [29].

In this study, SADRC based on a new SF is designed. The new SF can achieve the function of using LADRC under large perturbations but using NLADRC under small perturbations. It can adjust the parameters introduced in the SF to adjust that the error in what range then SADRC switching is performed. After that, the stability and convergence of SADRC with the new SF are demonstrated. Finally, the effectiveness of the proposed SADRC as a speed controller is verified through experiments.

The remainder of this paper is organized as follows. Section 2 provides a mathematical model of a PMSM. Section 3 introduces the principle of ADRC and then theoretically analyses the causes of the different performances between LADRC and NLADRC. Then, the proposed SADRC is given. Section 4 proves the convergence and stability of the proposed SADRC. Section 5 describes the experimental results and analysis of applying the proposed SADRC to a PMSM. Conclusions are drawn in Section 6.

## 2. Mathematical Model of a PMSM

The control object in this study is a PMSM. Assuming symmetrical windings and neglecting core saturation and disregarding eddy current losses and hysteresis losses, the mathematical model of a PMSM can be obtained according to the motor control theory in [30].

The stator voltage equations in the *d-q* synchronous rotating coordinate system are given as follows:

(1) $$u_{d}=Ri_{d}+\frac{d\psi_{d}}{dt}-\omega_{e}\psi_{q}$$

(2) $$u_{q}=Ri_{q}+\frac{d\psi_{q}}{dt}+\omega_{e}\psi_{d}$$

where $u_{q}$, $u_{d}$, $i_{q}$, and $i_{d}$ are the stator voltage and current in the *d-q* coordinate system, respectively. $\omega_{e}$ is the electric angular velocity, and *R* is the stator resistance. $\psi_{d}=L_{d}i_{d}+\psi_{f}$ and $\psi_{q}=L_{q}i_{q}$ are the stator flux linkages in the *d-q* coordinate system, where $L_{d}$ and $L_{q}$ are the inductances in the *d-q* coordinate system and $\psi_{f}$ is the flux amplitude of the permanent magnet.

The electromagnetic torque equation is expressed as follows:

(3) $$T_{e}=\frac{3}{2}n_{p}\left(\psi_{d}i_{q}-\psi_{q}i_{d}\right)$$

where $T_{e}$ is the electromagnetic torque and $n_{p}$ is the number of pole pairs.

The motion equation is as follows:

(4) $$J\frac{d\omega_{m}}{dt}=T_{e}-T_{L}-B\omega_{m}$$

where *J* is the moment of inertia, $\omega_{m}$ is the mechanical angular velocity, $T_{L}$ is the load torque, and *B* is the viscous friction coefficient.

## 3. Design of a Linear-Nonlinear Switching Active Disturbance Rejection Controller

### 3.1. Active Disturbance Rejection Control Algorithm

**Figure 1:**
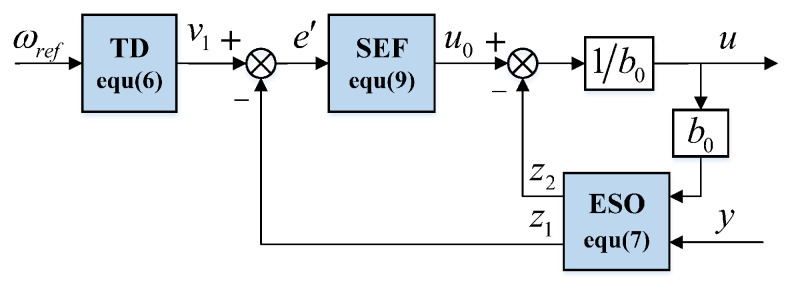
ADRC block diagram.

ADRC was proposed by Han [16]. It is a control algorithm with the function of estimating disturbances and compensating them in real time. ADRC consists of a TD, ESO, and state error feedback control law (SEF). Its block diagram is shown in Figure 1.

The control object in this study is a first-order system, so taking the first-order system as an example, the control object is expanded into a system of the following form according to the mathematical model of a PMSM.

(5) $$\left\{\begin{array}{c} {\dot{x}}_{1}=b_{0}\cdot u+x_{2}\\ {\dot{x}}_{2}=-h \end{array}\right.$$

where $u=i_{qref}$ is the input variable of the system, $i_{qref}$ is the reference value of $i_{q}$, $x_{1}=\omega_{m}$ and $x_{2}=-f+\left(b-b_{0}\right)i_{qref}$ are the state variables of the system, $b=\frac{3n_{p}\psi_{f}}{2J}$ is the control gain, $b_{0}$ is the estimated value of *b*, and $f(x_{i},\omega )$ is the total disturbance of the system, which consists of internal disturbance and external disturbance ω.

Since the system has inertia, the output variables of the system can only change slowly from zero initial states, while the initial value of the control variable is a nonzero variable reference value. Therefore, the larger the initial value of the control variable is, the larger the initial value of the system error, which causes a contradiction of rapidity and overshoot. To reduce this initial error and solve the contradiction between rapidity and overshoot, a TD is introduced as a transition process in ADRC, and its equation is as follows [16]:

(6) $$\left\{\begin{array}{c} {\dot{v}}_{1}=v_{2}\\ {\dot{v}}_{2}=-rsign\left(v_{1}-\omega_{ref}+\frac{v_{2}\left|v_{2}\right|}{2r}\right) \end{array}\right.$$

where $\omega_{ref}$ is the speed reference value, $v_{1}$ is the tracking value of $\omega_{ref}$, $v_{2}$ is the derivative of $v_{1}$ and *r* is the speed factor.

An ESO is an important part of ADRC. It can observe the internal and external disturbances affecting the controlled output in real time and compensate for the disturbances to eliminate the effects of the disturbances. Thus, ADRC has the function of anti-interference. The ESO equation is defined as follows:

(7) $$\left\{\begin{array}{c} {\dot{z}}_{1}=z_{2}-\beta_{1}\varphi_{1}\left(e\right)+b_{0}u\\ {\dot{z}}_{2}=-\beta_{2}\varphi_{2}\left(e\right) \end{array}\right.$$

where $e=z_{1}-y$ is the observation error of the ESO, $z_{i}$ is the estimate of the corresponding $x_{i}$, $\varphi_{i}\left(e\right)$ is a function of the observation error *e*, $\beta_{i}$ is the gain coefficient of the ESO, and i∈{1,2}.

The control law in (7) is defined as:

(8) $$u=\frac{u_{0}-z_{2}}{b_{0}}$$

where $u_{0}$ is the output variable of the SEF. For the first-order control object, its general form can be expressed as:

(9) $$u_{0}=k_{1}g\left(e^{\prime }\right)$$

where $k_{1}$ is the gain coefficient of the SEF, $e^{\prime }=v_{1}-z_{1}$ is the feedback error, and $g(e^{\prime })$ is a function of the feedback error $e^{\prime }$.

### 3.2. Linear-Nonlinear Switching Active Disturbance Rejection Control

ADRC can be divided into LADRC and NLADRC. The main difference between the two is the selection of the observation error function $\varphi_{i}\left(e\right)$ in the ESO and the feedback error function $g(e^{\prime })$ in the SEF.

In LADRC, $\varphi_{i}\left(e\right)=e,i\in \left\{1,2\right\}$, and $g\left(e^{\prime }\right)=e^{\prime }$. In NLADRC, $\varphi_{i}\left(e\right),i\in \left\{1,2\right\}$, and $g\left(e^{\prime }\right),i\in \left\{1,2\right\}$ are usually taken as nonlinear functions. A typical nonlinear function fal(x,α,δ) can be expressed as follows:

(10) $$fal\left(x,\alpha ,\delta \right)=\left\{\begin{array}{cc} \frac{x}{\delta^{1-\alpha }} & |x|\leq \delta \\ |x{|}^{\alpha }sign\left(x\right) & |x|>\delta \end{array}\right.$$

where α and δ are undetermined parameters, and usually α<1. In this case, the function fal has the characteristic of large error with a small gain and small error with a large gain. δ is the linear range to avoid the occurrence of minimal error with a maximum gain caused by the nonlinear function.

**Figure 2:**
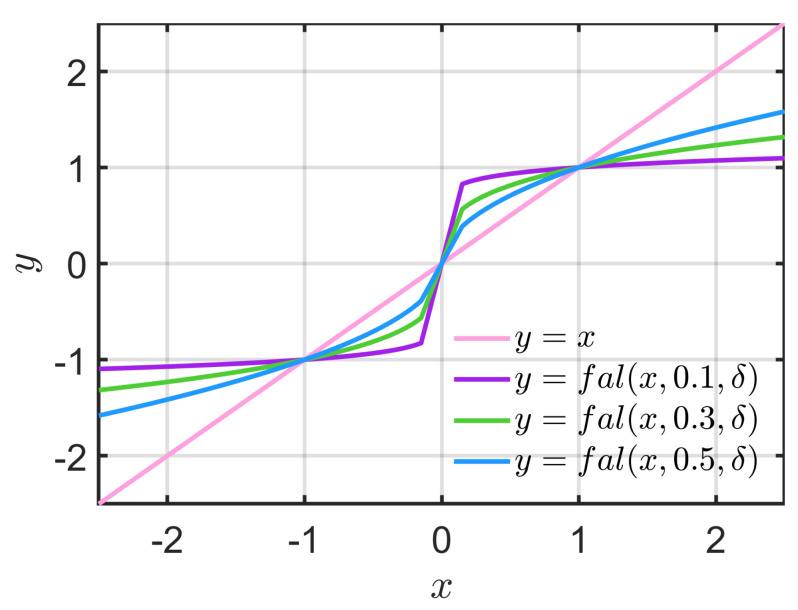
Comparison of the linear and nonlinear fal functions with different α values.

After studying [28,29], it was found through simulation and experimental results that LESO has the characteristics of easy parameter tuning and the anti-disturbance ability will change little with changing disturbance amplitude. In contrast, NLESO parameter tuning is relatively complicated, and the anti-disturbance ability is limited with increasing disturbance amplitude, but NLESO has better control precision. In other words, the performance of LESO is more advantageous under large error, while the performance of NLESO is more advantageous under small error. To explore the specific reasons for the different performances between LESO and NLESO, this study focuses on analysing the characteristics of the linear and nonlinear fal functions from the differences in their formulas. It was found that the performance of the nonlinear fal function changes with the values of its parameters. Among them, the function performance is more significantly influenced by the parameter α. The smaller α is, the higher the control precision of NLESO, but at the same time, the response speed will be slower. Figure 2 compares the linear and nonlinear fal functions with different α values. The comparison shows that as α decreases, the gain of function fal in the case of large errors decreases, which is the main reason for the slower response speed.

**Figure 3:**
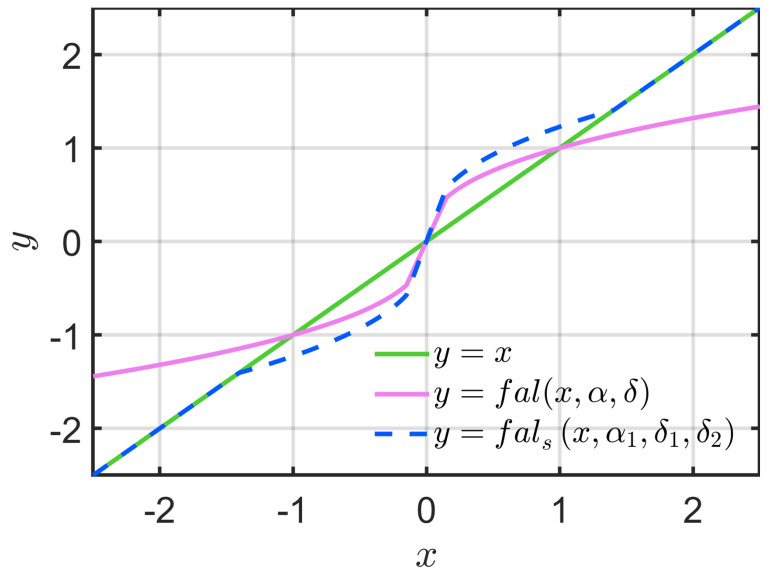
Comparison of the linear function, fal(x,α,δ) and $fal_{s}\left(x,\alpha_{1},\delta_{1},\delta_{2}\right)$.

To improve the contradiction between control precision and response speed, it is necessary to ensure that the control precision will not be degraded while improving the phenomenon that the gain of the function fal decreases with decreasing α in the case of large error. Therefore, this study combines linear and nonlinear functions to retain and improve their respective performance advantages and constructs an SF as follows:

(11) $$fal_{s}\left(x,\alpha_{1},\delta_{1},\delta_{2}\right)=\left\{\begin{array}{ll} \frac{x}{{\delta_{2}}^{\alpha_{1}}{\delta_{1}}^{1-\alpha_{1}}} & \left|x\right|\leq \delta_{1}\\ {\left|\frac{x}{\delta_{2}}\right|}^{\alpha_{1}}sign\left(x\right) & \delta_{1}<\left|x\right|<\delta_{2}^{\frac{\alpha_{1}}{\alpha_{1}-1}}\\ x & \left|x\right|\geq \delta_{2}^{\frac{\alpha_{1}}{\alpha_{1}-1}} \end{array}\right.$$

where $0<\alpha_{1}<1$, $0<\delta_{1}<\delta_{2}<1$. A comparison of the linear function,fal(x,α,δ) and $fal_{s}\left(x,\alpha_{1},\delta_{1},\delta_{2}\right)$ is shown in Figure 3. From Figure 3, the introduction of $\delta_{2}$ improves the gain in the nonlinear range. The introduction of $\delta_{2}$ also reduces the steady-state error of the ESO, as seen in the proof of SESO convergence in the next section, i.e., the introduction of the parameter $\delta_{2}$ can effectively improve the contradiction between the control precision and the response speed. In addition, the value of $\delta_{2}$ can be used to adjust the linear-nonlinear switching point of the function $fal_{s}\left(x,\alpha_{1},\delta_{1},\delta_{2}\right)$.

**Figure 4:**
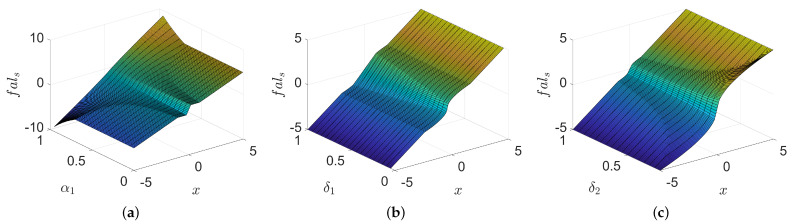
Effect of different parameter changes on $fal_{s}$. (**a**) Three-dimensional diagram of the effect of parameter $\alpha_{1}$. (**b**) Three-dimensional diagram of the effect of parameter $\delta_{1}$. (**c**) Three-dimensional diagram of the effect of parameter $\delta_{2}$.

In order to analyze the variation of $fal_{s}\left(x,\alpha_{1},\delta_{1},\delta_{2}\right)$ under the influence of various parameters more intuitively, the three-dimensional diagram shown in Figure 4 is given. Referring to (11) defines $\left|x\right|\leq \delta_{1}$ as linear region 1, defines $\delta_{1}<\left|x\right|<\delta_{2}^{\frac{\alpha_{1}}{\alpha_{1}-1}}$ as nonlinear region, defines $\left|x\right|\geq \delta_{2}^{\frac{\alpha_{1}}{\alpha_{1}-1}}$ as linear region 2. The linear-nonlinear switching point between linear region 1 and nonlinear region is defined as switching point 1, and the linear-nonlinear switching point between nonlinear region and linear region 2 is defined as switching point 2. From Figure 4a, we can see that $\alpha_{1}$ affects the position of the switching point 2, while affects the gain of the linear region 1 and the nonlinear region. With the increase of $\alpha_{1}$, the gain of linear region 1 decreases, the gain of nonlinear region becomes larger, and the value of switching point 2 also becomes larger. From Figure 4b, we can see that $\delta_{1}$ affects the position of switching point 1 and the gain of linear region 1 at the same time. As $\delta_{1}$ increases, the value of switching point 1 becomes larger and the gain of linear region 1 becomes smaller. From Figure 4c, it can be seen that $\delta_{2}$ affects the position of switching point 2, and affects the gain of both linear region 1 and nonlinear region. As $\delta_{2}$ increases, the value of switching point 2 decreases, and the gain of both linear region 1 and nonlinear region decreases.

**Figure 5:**
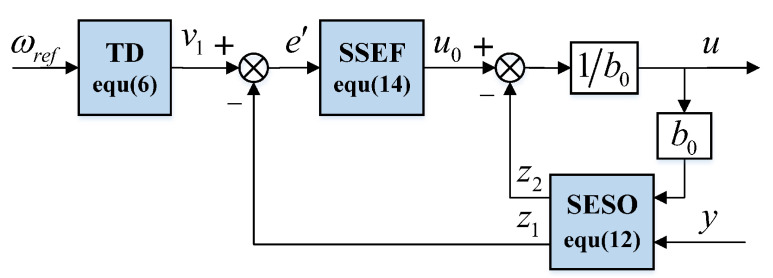
SADRC block diagram.

The newly constructed $fal_{s}$ function is applied to ESO and SEF to form SESO and SSEF, respectively, and the SADRC based on SESO and SSEF is proposed. Its structure is shown in Figure 5, where the expression of SESO is as follows:

(12) $$\left\{\begin{array}{c} {\dot{z}}_{1}=z_{2}-\beta_{1}e+b_{0}u\\ {\dot{z}}_{2}=-\beta_{2}fal_{s}\left(e,\alpha_{1},\delta_{1},\delta_{2}\right) \end{array}\right.$$

where $e=z_{1}-y$ is the observation error of SESO. The control law in (12) is defined as:

(13) $$u=\frac{u_{0}-z_{2}}{b_{0}}$$

where $u_{0}$ is the output variable of the linear-nonlinear switching state error feedback control law (SSEF). The SSEF in this study is a PI controller, and its expression is:

(14) $$u_{0}=k_{p}fal_{s}\left(e^{\prime },\alpha_{1},\delta_{1},\delta_{2}\right)+k_{i}\int_{0}^{e^{\prime }}fal_{s}\left(e^{\prime },\alpha_{1},\delta_{1},\delta_{2}\right)de^{\prime }$$

where $e^{\prime }=v_{1}-z_{1}$ is the feedback error of the SSEF and $k_{p}$ and $k_{i}$ are the gain coefficients of proportion and integration, respectively.

## 4. Stability and Convergence of Linear-Nonlinear Switching Controllers

### 4.1. Convergence of Linear-Nonlinear Switching Extended State Observer

To prove the convergence of SESO, the following assumptions are made.

**Assumption 1.**  *The total disturbance $f\left(x_{i},\omega \right),i\in \left\{1,2\right\}$ is continuous and derivable concerning its independent variable $x_{i}$, where ω is external disturbance.*

**Assumption 2.**  *h is the derivative of the total disturbance f along the trajectory, which satisfies $\begin{array}{cc} h_{0} & =\underset{t\in (0,+\infty )}{\sup}f\left({\dot{x}}_{i},\omega \right)<+\infty \end{array}$.*

**Theorem 1.**  *For the observation error system $e_{i}\left(t\right),i\in \left\{1,2\right\}$ of (12) and some positive definite function trajectory $V\left(e_{i}\right),i\in \left\{1,2\right\}$ about the error system there exist positive constants $\varepsilon_{1}>\varepsilon_{0}$, such that if $e_{i}\left(t\right)\in \Omega_{1}=\left\{e_{i}\left(t\right)|V\left(e_{i}\right)<\varepsilon_{1}\right\}$, then it will converge to the set $\Omega_{0}=\left\{e_{i}\left(t\right)|V\left(e_{i}\right)<\varepsilon_{0}\right\}$.*

**Proof.**  Equation (12) minus (5) yields the observation error system of SESO as follows:

(15) $$\left\{\begin{array}{c} {\dot{e}}_{1}=e_{2}-\beta_{1}e_{1}\\ {\dot{e}}_{2}=h-\beta_{2}fal_{s}\left(e_{1},\alpha_{1},\delta_{1},\delta_{2}\right) \end{array}\right.$$

For convenience of presentation, $fal_{s}\left(x,\alpha_{1},\delta_{1},\delta_{2}\right)$ is abbreviated as $fal_{s}\left(x\right)$ in the subsequent proof. For the error system (15), a linear transformation of the following form is performed:

(16) $$\left\{\begin{array}{c} \eta_{1}=e_{1}\\ \eta_{2}=e_{2}-\beta_{1}e_{1} \end{array}\right.$$

Then, the system equivalent to (15) is obtained as follows:

(17) $$\left\{\begin{array}{c} {\dot{\eta }}_{1}=\eta_{2}\\ {\dot{\eta }}_{2}=h-\beta_{2}fal_{s}\left(\eta_{1}\right)-\beta_{1}\eta_{2} \end{array}\right.$$

The equivalent system and the original system have the same set of zeros and poles, so the equivalent system has the same convergence as the original system [31]. Therefore, the Lyapunov function of (17) is constructed as follows:

(18) $$V\left(\eta \right)=V_{1}\left(\eta_{1}\right)+V_{2}\left(\eta_{2}\right)$$

where

(19) $$\begin{array}{cc} V_{1}\left(\eta_{1}\right) & =\beta_{2}\int_{0}^{\eta_{1}}fal_{s}\left(\eta_{1}\right)d\eta_{1}\\ & =\left\{\begin{array}{cc} \int_{0}^{\eta_{1}}\beta_{2}ab\eta_{1}d\eta_{1} & \left|\eta_{1}\right|\leq \delta_{1}\\ \int_{0}^{\eta_{1}}\beta_{2}b{\left|\eta_{1}\right|}^{\alpha_{1}}\mathrm{sign}\left(\eta_{1}\right)d\eta_{1} & \delta_{1}<\left|\eta_{1}\right|<\delta_{2}^{\frac{\alpha_{1}}{\alpha_{1}-1}}\\ \int_{0}^{\eta_{1}}\beta_{2}\eta_{1}d\eta_{1} & \left|\eta_{1}\right|\geq \delta_{2}^{\frac{\alpha_{1}}{\alpha_{1}-1}} \end{array}\right. \end{array}$$

(20) $$V_{2}\left(\eta_{2}\right)=\frac{1}{2}\eta_{2}^{2}$$

The parameters in $V_{1}\left(\eta_{1}\right)$ satisfy $a=\frac{1}{\delta_{1}^{1-\alpha_{1}}}$, $b=\frac{1}{\delta_{2}^{\alpha_{1}}}$. The derivative of V(η) is

(21) $$\dot{V}\left(\eta \right)=\frac{\partial V}{\partial \eta_{1}}{\dot{\eta }}_{1}+\frac{\partial V}{\partial \eta_{2}}{\dot{\eta }}_{2}=\eta_{2}\left(h-\beta_{1}\eta_{2}\right)$$

By mathematical derivation, if $\left|\eta_{2}\right|>\frac{h_{0}}{\beta_{1}}$ can guarantee $\dot{V}\left(\eta \right)<0$, i.e., the trajectories of the system will eventually enter the range $\left|\eta_{2}\right|\leq \frac{h_{0}}{\beta_{1}}$, $\frac{h_{0}}{\beta_{1}}$ is the error bound for $\eta_{2}$. Substituting (11) into (17), the formula of ${\dot{\eta }}_{2}$ is obtained as follows:

(22) $${\dot{\eta }}_{2}=\left\{\begin{array}{ll} h-\beta_{2}ab\eta_{1}-\beta_{1}\eta_{2} & \left|\eta_{1}\right|\leq \delta_{1}\\ h-\beta_{2}b{\left|\eta_{1}\right|}^{\alpha_{1}}sign\left(\eta_{1}\right)-\beta_{1}\eta_{2} & \delta_{1}<\left|\eta_{1}\right|<\delta_{2}^{\frac{\alpha_{1}}{\alpha_{1}-1}}\\ h-\beta_{2}\eta_{1}-\beta_{1}\eta_{2} & \left|\eta_{1}\right|\geq \delta_{2}^{\frac{\alpha_{1}}{\alpha_{1}-1}} \end{array}\right.$$

On the $\eta_{1}$ axis, i.e., $\eta_{2}=0$, we can obtain

(23) $${\dot{\eta }}_{2}=\left\{\begin{array}{ll} h-\beta_{2}ab\eta_{1} & \left|\eta_{1}\right|\leq \delta_{1}\\ h-\beta_{2}b{\left|\eta_{1}\right|}^{\alpha_{1}}sign\left(\eta_{1}\right) & \delta_{1}<\left|\eta_{1}\right|<\delta_{2}^{\frac{\alpha_{1}}{\alpha_{1}-1}}\\ h-\beta_{2}\eta_{1} & \left|\eta_{1}\right|\geq \delta_{2}^{\frac{\alpha_{1}}{\alpha_{1}-1}} \end{array}\right.$$

When the system reaches the equilibrium point, i.e., ${\dot{\eta }}_{2}=0$, the equilibrium point of the system (17) in the range $\left|\eta_{1}\right|\leq \delta_{1}$ is

$$\begin{array}{c} \eta_{1}=\frac{h}{\beta_{2}ab},\eta_{2}=0 \end{array}$$

□

According to Assumption 2, $\left|\eta_{1}\right|\leq \frac{h_{0}}{\beta_{2}ab}$, which proves that the steady-state error of system (17) in the range $\left|\eta_{1}\right|\leq \delta_{1}$ will eventually converge to the range $\left|\eta_{1}\right|\leq \frac{h_{0}}{\beta_{2}ab}$, $\left|\eta_{2}\right|\leq \frac{h_{0}}{\beta_{1}}$.

Similarly, it can be calculated that when $\left|e_{1}\right|\leq \delta_{1}$, the steady-state error of NLESO applying the nonlinear function (10) is $\left|e_{1}\right|\leq \frac{h_{0}}{\beta_{2}a_{0}},\left|e_{2}\right|\leq \frac{h_{0}}{\beta_{1}}$, where $a_{0}=\frac{1}{\delta^{1-\alpha }}$, let $\alpha =\alpha_{1}$, then $a=a_{0}$. Since $b=\frac{1}{\delta_{2}^{\alpha_{1}}}>1,\delta_{2}$ reduces the steady-state error of SESO and improves the control precision.

When $\delta_{1}<\left|\eta_{1}\right|<\delta_{2}^{\frac{\alpha_{1}}{\alpha_{1}-1}}$, the equilibrium point of system (17) is

$$\begin{array}{c} \eta_{1}={\left(\frac{\left|h\right|}{\beta_{2}b}\right)}^{\frac{1}{\alpha_{1}}}sign\left(\eta_{1}\right),\eta_{2}=0 \end{array}$$

According to Assumption 2, $\left|\eta_{1}\right|\leq {\left(\frac{h_{0}}{\beta_{2}b}\right)}^{\frac{1}{\alpha_{1}}}$, which proves that the steady-state error of system (17) in the range $\delta_{1}<\left|\eta_{1}\right|<\delta_{2}^{\frac{\alpha_{1}}{\alpha_{1}-1}}$ will eventually converge to the range $\left|\eta_{1}\right|\leq {\left(\frac{h_{0}}{\beta_{2}b}\right)}^{\frac{1}{\alpha_{1}}},\left|\eta_{2}\right|\leq \frac{h_{0}}{\beta_{1}}$.

Similarly, it can be calculated that when $\left|e_{1}\right|>\delta_{1}$, the steady-state error of NLESO applying the nonlinear function (10) is $\left|e_{1}\right|\leq {\left(\frac{h_{0}}{\beta_{2}}\right)}^{\frac{1}{\alpha }},\left|e_{2}\right|\leq \frac{h_{0}}{\beta_{1}}$. Since $b=\frac{1}{\delta_{2}^{\alpha_{1}}}>1,\delta_{2}$ reduces the steady-state error of SESO and improves the control precision.

When $\left|\eta_{1}\right|\geq \delta_{2}^{\frac{\alpha_{1}}{\alpha_{1}-1}}$, the equilibrium point of system (17) is

$$\begin{array}{c} \eta_{1}=\frac{h}{\beta_{2}},\eta_{2}=0 \end{array}$$

According to Assumption 2, $\left|\eta_{1}\right|\leq \frac{h_{0}}{\beta_{2}}$, which proves that the steady-state error of system (17) in the range $\left|\eta_{1}\right|\geq \delta_{2}^{\frac{\alpha_{1}}{\alpha_{1}-1}}$ eventually converges to the range $\left|\eta_{1}\right|\leq \frac{h_{0}}{\beta_{2}},\left|\eta_{2}\right|\leq \frac{h_{0}}{\beta_{1}}$.

**Figure 6:**
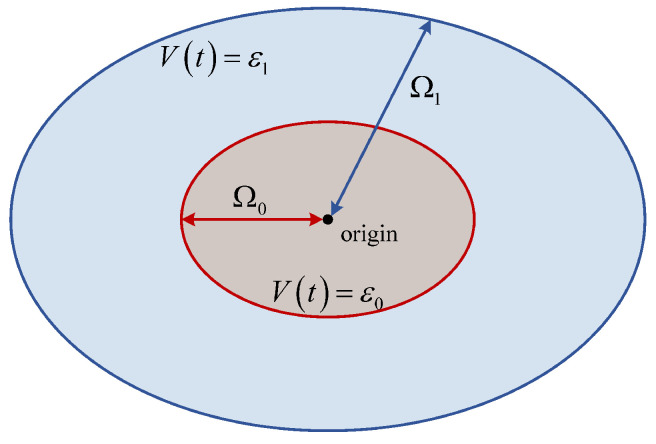
Boundary of the observation error.

In summary, according to the Lyapunov stability theorem and its implications, when the error is not zero, taking the positive definite function $V(\eta_{i})$ in the form of (18), there exist sets $\Omega_{1}$ and $\Omega_{0}$ satisfying the condition such that if the estimation error $\eta_{i}\left(t\right)\in \Omega_{1}-\Omega_{0}$, then $\dot{V}\left(\eta_{i}\right)<0$. That is, $\eta_{i}$ in the set $\Omega_{1}$ will gradually converge to the set $\Omega_{0}$ along the trajectory $V(\eta_{i})$, as shown in Figure 6, i.e., Theorem 1 is proved. The above proof process also gives the final steady-state error convergence range of the error system, and it can be seen that $\delta_{2}$ reduces the steady-state error convergence range and improves the control precision.

### 4.2. Closed-Loop Stability

According to the controller Equations (13) and (14), the SSEF controller is composed as follows:

(24) u=kpfalse′,α1,δ1,δ2+ki∫0e′false′,α1,δ1,δ2de′−z2kpfalse′,α1,δ1,δ2+ki∫0e′false′,α1,δ1,δ2de′−z2b0b0

Let

(25) $$s_{1}=v_{1}-x_{1}$$

In (25), the derivative of $s_{1}$ is:

(26) $${\dot{s}}_{1}={\dot{v}}_{1}-\left(b_{0}u+x_{2}\right)$$

where ${\dot{v}}_{1}$ is continuous and bounded. According to the errors (15) and (25) can be easily obtained $e^{\prime }=s_{1}-e_{1}$. In addition there are

(27) $${\dot{s}}_{1}={\dot{v}}_{1}-k_{p}fal_{s}\left(e^{\prime },\alpha_{1},\delta_{1},\delta_{2}\right)-k_{i}\int_{0}^{e^{\prime }}fal_{s}\left(e^{\prime },\alpha_{1},\delta_{1},\delta_{2}\right)de^{\prime }+e_{2}$$

where, $e_{2}=z_{2}-f$.

**Theorem 2.**  *There exists appropriate positive coefficient $k_{p}$ and $k_{i}$, which makes the feedback error closed-loop system stable under the control of the controller (24).*

**Proof.**  The Lyapunov function is constructed as follows:

(28) $$\begin{array}{cc} V\left(e^{\prime }\right) & =\frac{1}{2}{e^{\prime }}^{2}\\ \; & =\frac{1}{2}{\left(s_{1}-e_{1}\right)}^{2} \end{array}$$

The derivative of $V(e^{\prime })$ is

(29) $$\begin{array}{cc} \dot{V}\left(e^{\prime }\right) & =e^{\prime }{\dot{e}}^{\prime }\\ \; & =e^{\prime }\left({\dot{v}}_{1}-k_{p}fal_{s}\left(e^{\prime },\alpha_{1},\delta_{1},\delta_{2}\right)-k_{i}\int_{0}^{e^{\prime }}fal_{s}\left(e^{\prime },\alpha_{1},\delta_{1},\delta_{2}\right)de^{\prime }+e_{2}-{\dot{e}}_{1}\right)\\ \; & =-e\left(k_{p}fal_{s}\left(e^{\prime },\alpha_{1},\delta_{1},\delta_{2}\right)+k_{i}\int_{0}^{e^{\prime }}fal_{s}\left(e^{\prime },\alpha_{1},\delta_{1},\delta_{2}\right)de^{\prime }\right)+\left(s_{1}-e_{1}\right)\left({\dot{v}}_{1}+\beta_{2}e_{1}\right) \end{array}$$

It is worth noting that $-e\left(k_{p}fal_{s}\left(e^{\prime },\alpha_{1},\delta_{1},\delta_{2}\right)+k_{i}\int_{0}^{e^{\prime }}fal_{s}\left(e^{\prime },\alpha_{1},\delta_{1},\delta_{2}\right)de^{\prime }\right)\leq 0$. By Theorem 1, we know that both $e_{1}$ and $e_{2}$ are bound. Moreover, $s_{1}$ is also bounded in practice. Letting $M_{1}=\left|\left(s_{1}-e_{1}\right)\left({\dot{v}}_{1}+\beta_{2}e_{1}\right)\right|$, one obtains that $M_{1}$ is also bounded, and therefore, rewriting (29) yields $\dot{V}\left(e^{\prime }\right)\leq -e\left(k_{p}fal_{s}\left(e^{\prime },\alpha_{1},\delta_{1},\delta_{2}\right)+k_{i}\int_{0}^{e^{\prime }}fal_{s}\left(e^{\prime },\alpha_{1},\delta_{1},\delta_{2}\right)de^{\prime }\right)+M_{1}$. Therefore, there exists suitable $k_{p}$ and $k_{i}$ to ensure $\dot{V}\left(e^{\prime }\right)\leq 0$. In summary, when $\dot{V}\left(e^{\prime }\right)\leq 0$, $V(e^{\prime })$ is a positive definite function, and there exists suitable $k_{p}$ and $k_{i}$ to ensure its derivative $V(e^{\prime })$ is negative definite, which satisfies Lyapunov’s stability theorem, i.e., Theorem 2 is proved. □

## 5. Experimental Results and Discussion

### 5.1. Experimental Platform

**Figure 7:**
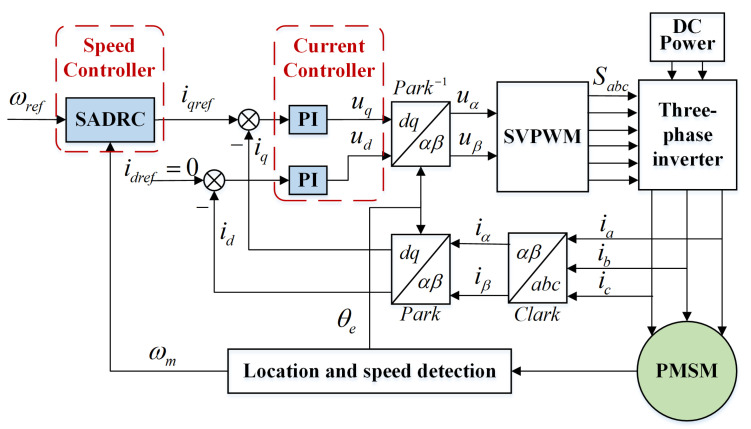
Structure diagram of the PMSM servo system based on SADRC.

To further verify the performance of the SADRC proposed in this paper, experimental verification is performed on a 707 W PMSM drive platform. Figure 7 shows the overall structure of the PMSM servo system with the application of SADRC. The field-oriented control (FOC) method is used to control the PMSM, SADRC is used as the speed controller to output the reference current $i_{qref}$ of the current loop, and PI is used as the current controller to output the control voltage $u_{q}$.

**Figure 8:**
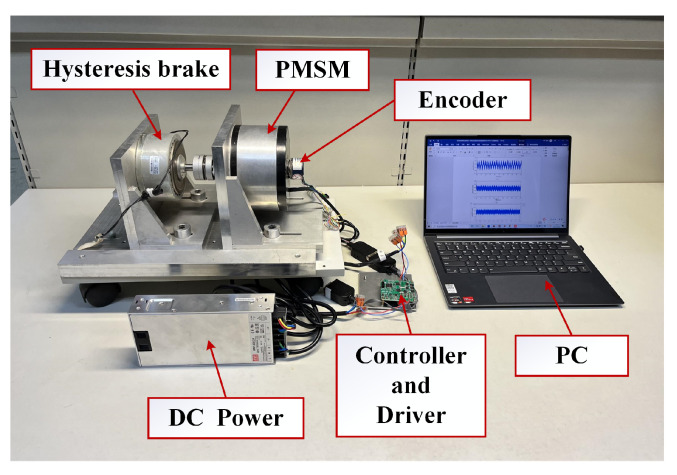
Photograph of the experimental platform.

The 707 W PMSM driver platform is shown in Figure 8, and the corresponding technical parameters of the PMSM are shown in Table 1. A hysteresis brake is used to generate the load torque. An absolute encoder is installed at the end of the shaft to measure the digital position to obtain the speed of the PMSM. The core component of the controller is a DSP-TMS320F280049, and the control algorithms are implemented in digital signal processing (DSP) using a C program. The core components of the driver are a DRV8350 three-phase smart gate driver and a power MOSFET.

### 5.2. Parameter Tuning

The only parameter that needs to be tuned in the TD is the speed factor *r*. The value of *r* directly affects the tracking speed of the TD. The larger *r* is, the faster $v_{1}$ will be to keep up with $\omega_{ref}$. Finally, $r=10^{5}$.

There are two types of parameters to be tuned in the ESO: the gain $\beta_{i}$ and the parameters in the functions fal(x,α,δ), $fal_{s}\left(x,\alpha_{1},\delta_{1},\delta_{2}\right)$. The gain $\beta_{i}$ is selected by referring to the idea of determining the ESO parameters with the concept of bandwidth proposed by Gao [18], and Equation (7) is rewritten in the following form:

(30) $$\left\{\begin{array}{c} {\dot{z}}_{1}=z_{2}-\beta_{1}\lambda_{1}\left(e\right)e+b_{0}u\\ {\dot{z}}_{2}=-\beta_{2}\lambda_{2}\left(e\right)e \end{array}\right.$$

where $\lambda_{1}\left(e\right)=\frac{\varphi_{1}\left(e\right)}{e},\lambda_{2}\left(e\right)=\frac{\varphi_{2}\left(e\right)}{e}$. The transfer function of the disturbance observation $z_{2}$ is

(31) $$z_{2}=\frac{\beta_{2}\lambda_{2}\left(e\right)sy-\beta_{2}\lambda_{2}\left(e\right)b_{0}u}{s^{2}+\beta_{1}\lambda_{1}\left(e\right)s+\beta_{2}\lambda_{2}\left(e\right)}$$

The ESO can well suppress the perturbation of *u*. Meanwhile, to simplify the analysis, ignoring the influence of *u* and $\lambda_{i}\left(e\right)$, the denominator of (31) is formulated to ${\left(s+\omega_{0}\right)}^{2}$, which can make the second-order system ESO better observe the perturbation. That is, $\beta_{1}=2\omega_{0}$, $\beta_{2}=\omega_{0}^{2}$, and $\omega_{0}$ is the bandwidth of the ESO. Finally, according to the experimental requirements, $\omega_{0}=100$ rad/s, i.e., $\beta_{1}=200$, $\beta_{2}=10^{4}$. The values of α and $\alpha_{1}$ directly affect the gain and control precision. The smaller α and $\alpha_{1}$ are, the smaller the gain in the nonlinear range will be, and the more likely it is to cause high-frequency oscillation at the same time, but the control precision will become higher. δ and $\delta_{1}$ are the linear ranges of the function. $\delta_{2}$ affects the position of the linear-nonlinear switching point of the function $fal_{s}$. Considered comprehensively, α=0.5, δ=0.03, $\alpha_{1}=0.5$, $\delta_{1}=0.03$, and $\delta_{2}=0.5$.

The SEF uses the PI controller, and the parameters to be tuned are the gain $k_{p},k_{i}$ and the parameters in the functions fal(x,α,δ) and $fal_{s}\left(x,\alpha_{1},\delta_{1},\delta_{2}\right)$. The parameters in the functions fal(x,α,δ) and $fal_{s}\left(x,\alpha_{1},\delta_{1},\delta_{2}\right)$ are tuned in a similar way to the parameters in the ESO. Finally, $k_{p}=18$, $k_{i}=6$, α=0.5, δ=0.03, $\alpha_{1}=0.5$, $\delta_{1}=0.03$, and $\delta_{2}=0.5$.

$b_{0}$ is the estimated value of *b*. According to the parameters of the PMSM, b=208 is calculated. However, in the actual experiment, the value of *b* changes in real time due to model uncertainty, the perturbation of motor parameters, etc. The value of $b_{0}$ reflects the compensation capability of ADRC; the smaller $b_{0}$ is, the faster the disturbance compensation response, but it is also more likely to cause overshoot and oscillation of the disturbance observation. Considered comprehensively, $b_{0}=0.5b=104$.

### 5.3. Speed Step Experiment

**Figure 9:**
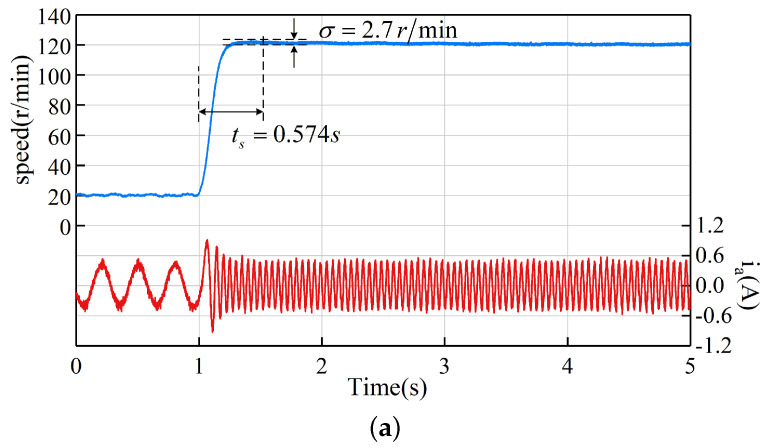

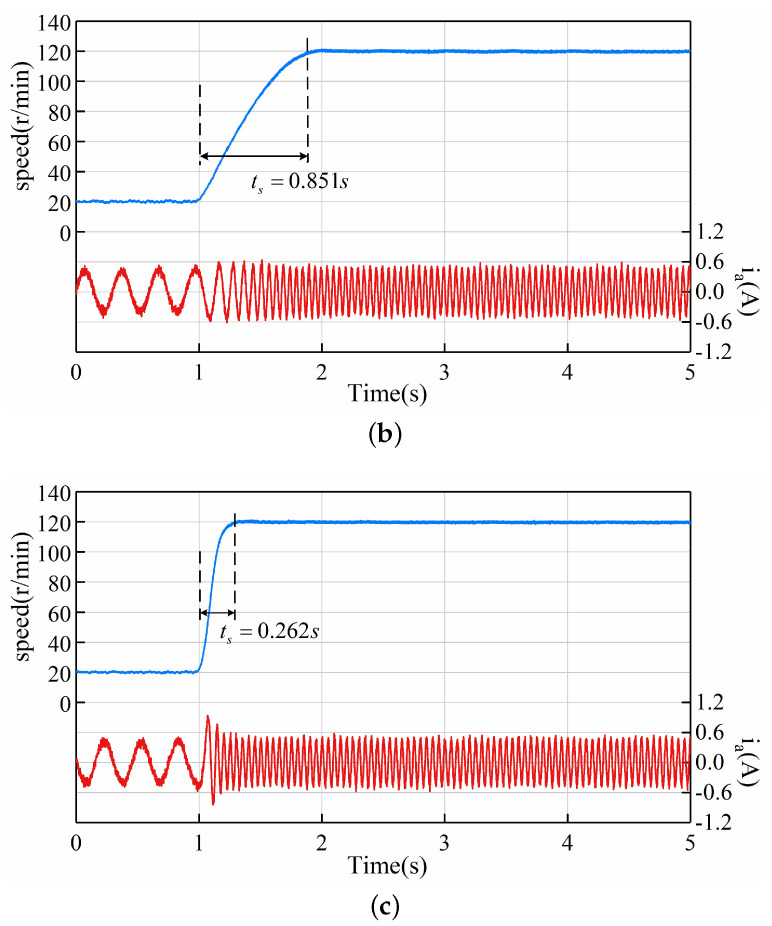
Speed step experiment from 20 r/min to 120 r/min: (**a**) LADRC (**b**) NLADRC (**c**) SADRC.

The first experiment compares the performance in the case of a speed step change. The speed $\omega_{m}$ response curves and phase current $i_{a}$ waveforms of the LADRC, NLADRC, and SADRC are given in Figure 9 for the case where the reference speed $\omega_{ref}$ changes from 20 r/min to 120 r/min at 1 s. The σ labelled in the figure is the speed overshoot, and $t_{s}$ is the adjustment time. The speed overshoot and adjustment time of the three algorithms are shown in Table 2. The experimental results show that LADRC has a speed overshoot of 2.7 r/min and a tuning time of 0.574 s, which is less than NLADRC, which has almost no overshoot, but its adjustment time is 0.851 s, slower than LADRC. The SADRC control strategy combines the advantages of both LADRC and NLADRC. It has almost no overshoot and a faster adjustment time of 0.262 s.

### 5.4. Steady-State Performance

**Figure 10:**
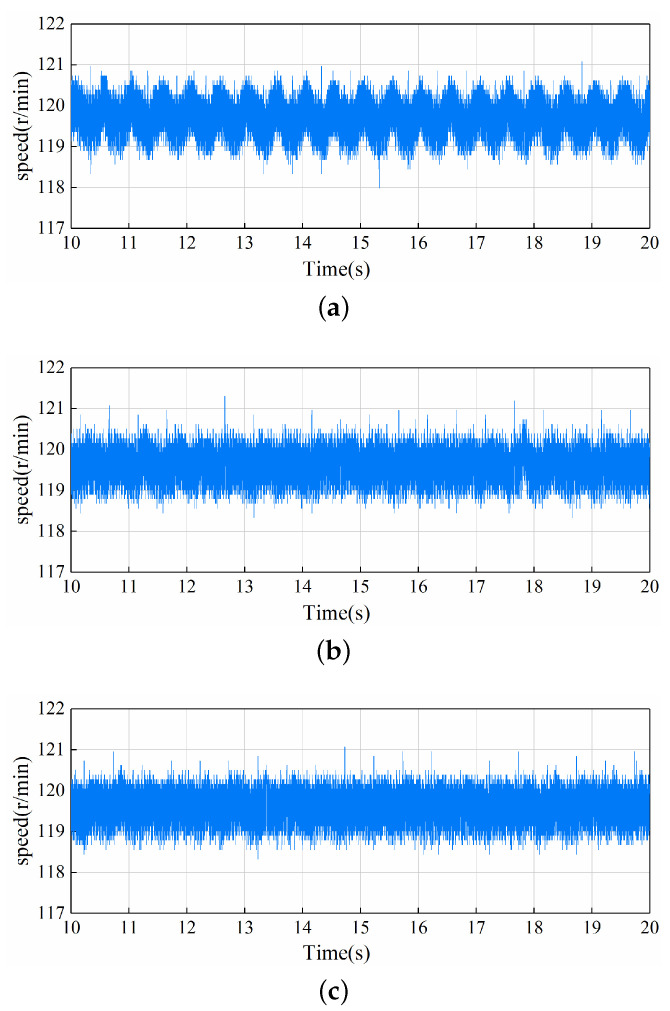
Speed waveforms at steady-state for the three control strategies: (**a**) LADRC (**b**) NLADRC (**c**) SADRC.

The second experiment compares the speed response at steady-state. The speed waveforms of the LADRC, NLADRC, and SADRC control strategies at steady-state are shown in Figure 10. It can be clearly seen that the LADRC control strategy has a larger speed fluctuation under steady-state conditions, and NLADRC and SADRC have the weakest speed fluctuation in comparison. Table 3 gives the maximum speed, minimum speed and range of the steady-state waveforms of LADRC, NLADRC, and SADRC shown in Figure 10 to measure the steady-state performance of the three control strategies. The range of LADRC, NLADRC, and SADRC are 3.090 r/min, 2.975 r/min, and 2.747 r/min. As can be seen from the figures and tables, SADRC has the most stable speed waveform in the steady-state case, followed by NLADRC, then LADRC.

### 5.5. Step Load Experiment

**Figure 11:**
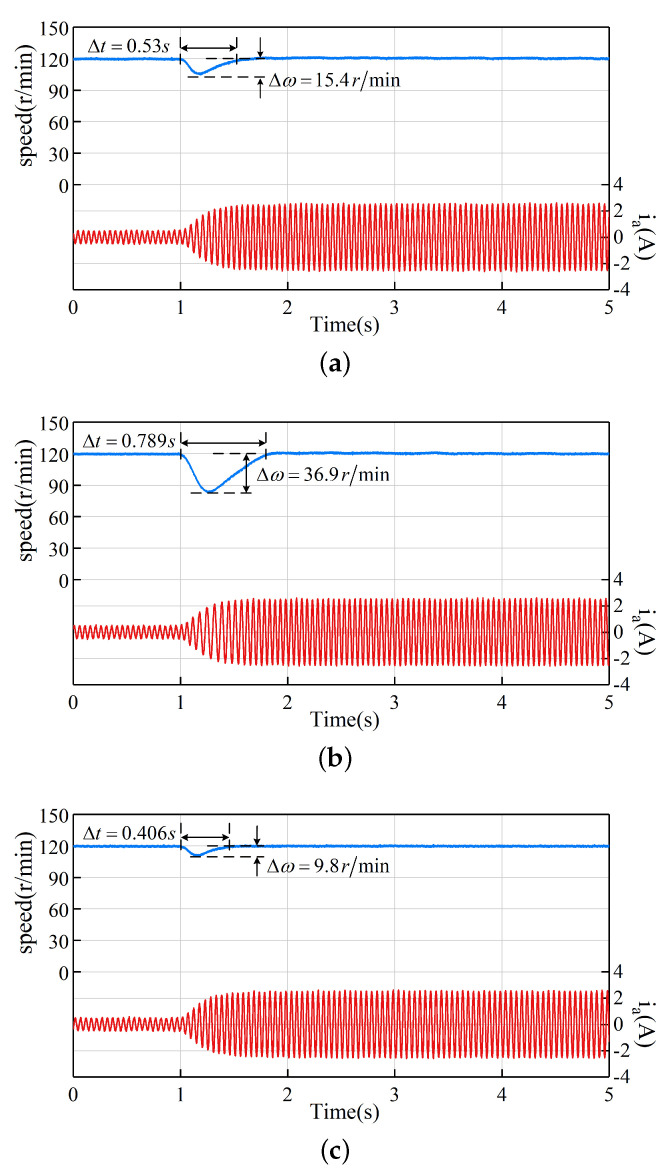
Experiment of the three control strategies under a step load of 1 N·m: (**a**) LADRC (**b**) NLADRC (**c**) SADRC.

The third experiment compares the anti-disturbance performance of the LADRC, NLADRC, and SADRC control strategies under step load disturbance. Figure 11 shows the waveforms of the speed $\omega_{m}$ and phase current $i_{a}$ at a speed of 120 r/min with a step load torque of 1 N·m. The maximum speed fluctuation Δω and the adjustment time Δt are labelled in Figure 11, and the maximum speed fluctuation and the adjustment time of the three algorithms are shown in Table 4. The maximum speed fluctuations of LADRC, NLADRC and SADRC are 15.4 r/min, 36.9 r/min and 9.8 r/min, respectively, and the adjustment times are 0.530 s, 0.789 s and 0.406 s, respectively. From the experimental results, it can be concluded that the NLADRC has the largest speed fluctuation and the longest adjustment time under a step load disturbance of 1 N·m, followed by LADRC, and finally SADRC. In other words, SADRC has the best anti-disturbance performance among the three control strategies. Between LADRC and NLADRC, LADRC has better anti-disturbance performance than NLADRC under a 1 N·m step load disturbance.

### 5.6. Sinusoidal Signal Tracking Experiment

**Figure 12:**
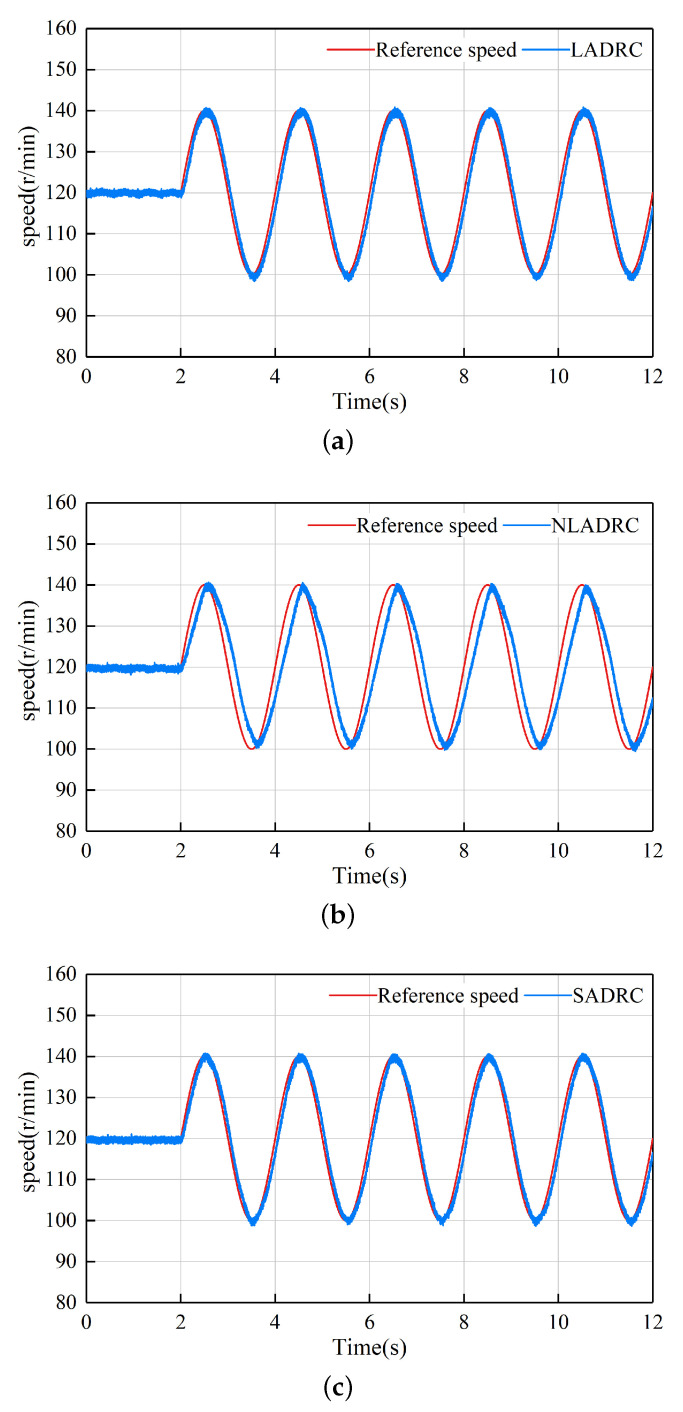
Tracking waveforms of the three control strategies under sinusoidal signal: (**a**) LADRC (**b**) NLADRC (**c**) SADRC.

The fourth experiment compares the tracking performance of LADRC, NLADRC, and SADRC algorithms under sinusoidal reference signals. Figure 12 shows the comparison of speed waveform and reference waveform under the control of the four algorithms. From the experimental results, the tracking signal of NLADRC is more accurate in terms of amplitude, but there is a tracking time delay. The tracking capability of LADRC and SADRC is relatively strong. Under the control of LADRC and SADRC, the tracking effect of motor speed is closest to the reference speed.

### 5.7. Comprehensive Comparison

**Figure 13:**
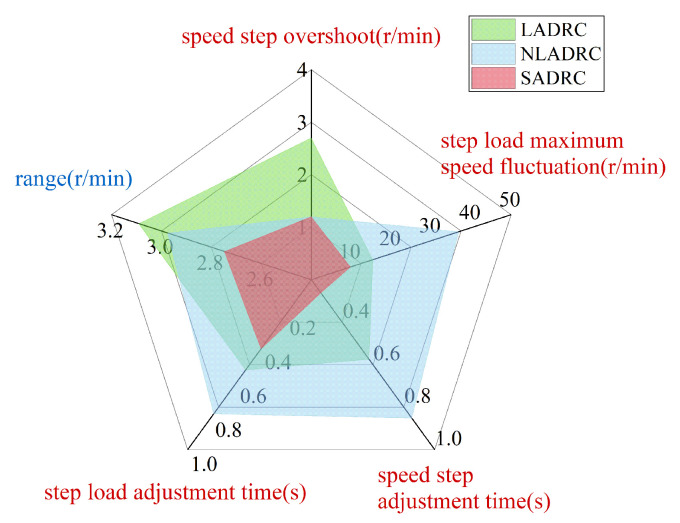
Comprehensive performance comparison of the LADRC, NLADRC and SADRC.

Figure 13 shows a comprehensive comparison of the LADRC, NLADRC, and SADRC under the three experimental conditions. The speed overshoot and adjustment time in the speed step experiment, the range in the steady-state experiment, and the maximum speed fluctuation and adjustment time in the step load experiment are selected as the comparison items. Among them, the comparison items marked in red can be regarded as the dynamic performance reference index of the algorithms, and the comparison items marked in blue can be regarded as the steady-state performance reference index of the algorithms. From the comprehensive comparison in Figure 13, it is clear that LADRC has better dynamic performance than NLADRC, and NLADRC has better steady-state performance than LADRC. SADRC has both the performance advantages of LADRC and NLADRC, and its performance is improved in most aspects compared with LADRC and NLADRC. Therefore, the SADRC control strategy proposed in this paper is feasible and effective.

## 6. Conclusions

In this paper, SADRC based on an SF is proposed. The novel SF constructed in this study can adjust the switching point by adjusting the value of the newly introduced parameter $\delta_{2}$. The SADRC algorithm can effectively combine the LADRC advantages of fast response and anti-disturbance performance that are not limited by the increase of disturbance amplitude and the NLADRC advantages of high accuracy. The SADRC control strategy is comprehensively compared with LADRC, and NLADRC on a 707 W PMSM platform. The experimental results show that SADRC has the smallest speed fluctuation and the least adjustment time compared with the other two control strategies under step change of speed and applied step load disturbance. Under steady-state conditions, SADRC has the most stable speed fluctuation. The SADRC also has superior tracking performance in sinusoidal tracking experiments. Therefore, the proposed SADRC better combines and improves the performance advantages of LADRC and NLADRC, and its feasibility and effectiveness have been verified.

## Figures and Tables

**Table 1:** PMSM parameter.

Symbol	Description	Value
*P*	Rate power	707 W
*R*	Armature resistanc	0.12 Ω
$L_{d}$	Inductance of *d* axis	0.2 mH
$L_{q}$	Inductance of *q* axis	0.2 mH
$K_{t}$	Torque coefficient	0.46 Nm/A
$n_{p}$	Number of pole pairs	10
*J*	Inertia	$221\times 10^{-5}\;\mathrm{Kg}\cdot m^{2}$

**Table 2:** Performance comparison of speed step experiment.

	LADRC	NLADRC	SADRC
Speed overshoot (r/min)	2.7	0	0
Adjustment time (s)	0.574	0.851	0.262

**Table 3:** Performance comparison of steady-state experiment.

	LADRC	NLADRC	SADRC
Maximum speed (r/min)	121.079	121.307	121.079
Minimum speed (r/min)	117.989	118.332	118.332
Range (r/min)	3.090	2.975	2.747

**Table 4:** Performance comparison of step load experiment.

	LADRC	NLADRC	SADRC
Maximum speed fluctuation (r/min)	15.4	36.9	9.8
Adjustment time (s)	0.530	0.789	0.406

## Data Availability

Not applicable.
